# Supplementary material for: Ecological Effects and Microbial Regulatory Mechanisms of Functional Grass Species Assembly in the Restoration of “Heitutan” Degraded Alpine Grasslands
Source: Microorganisms. 2025 Oct 11;13(10):2341. doi: 10.3390/microorganisms13102341 (PMC12566440; doi:10.3390/microorganisms13102341)
Supplement: Supplementary file 1 [file microorganisms-13-02341-s001.zip › microorganisms-3831276-supplementary.pdf]

Table 1 Calculation Table of Bacterial α Diversity

Estimators	HA-Mean	HB-Mean	HC-Mean	HD-Mean	HE-Mean	HF-Mean	The proportion of HE is higher than that of HA	The proportion of HE is lower than that of HA	The proportion of HD is lower than that of HA
OTUs	1648	1825.7	1839	1882.3	1967	1872	19.36%		
shannon	6.1737	6.2234	6.2745	6.3482	6.43	6.3322	4.20%		
simpson	0.0053	0.0054	0.005	0.0048	0.0043	0.0051		17.95%	8.50%
ace	1787.2	1930.9	1955.3	1990.9	2056	1974.8	15.04%		
Pielou_e	0.8335	0.8287	0.8347	0.8419	0.85	0.8404	1.76%		
chao1	1837.4	1955	1990.1	2022.2	2083	1994.7	13.37%		

Table 2 Calculation of Relevant Data of Bacterial Neural Network

Treatment	Edge count	Positive correlation ratio	Number of network nodes
HA	578	50.69%	49
HB	394	47.46%	50
HC	548	57.30%	50
HD	422	50.71%	50
HE	396	48.99%	49
HF	392	56.12%	50
